# Highlights from Heart Rhythm 2019: Cardiac Ablation, Pacing, and Monitoring

**DOI:** 10.19102/icrm.2019.100806

**Published:** 2019-08-15

**Authors:** Claudio Tondo

**Affiliations:** ^1^Heart Rhythm Center, Monzino Cardiac Center, Department of Clinical Sciences and Community Health, University of Milan, Milan, Italy; ^2^Texas Cardiac Arrhythmia Institute, Austin, TX, USA; ^3^Vrije Universiteit Brussel (VUB), Brussel, Belgium

**Keywords:** Atrial fibrillation, cardiac monitoring, catheter ablation, His bundle

The annual congress of the Heart Rhythm Society (HRS) held earlier this year in San Francisco drew attendees from around the world, confirming the high value of the conference and the prominent role of the congress in the international cardiac electrophysiology community. Upon browsing the program, it appeared that there was a resurgent interest in gaining a better understanding of the different potential mechanisms underlying both paroxysmal and persistent atrial fibrillation (AF) and which ablation strategies should be chosen to achieve an effective outcome.

Besides this, an overwhelming amount of data were presented on the subject of novel ablative approaches for both AF and ventricular tachycardia (VT), thus heralding an expected future improvement also in the treatment of the most complex cardiac arrhythmias.

In this commentary, I would like to emphasize certain aspects related to the newest ablative techniques and address particular topics in the field of cardiac pacing and upcoming new modalities of remote cardiac rhythm monitoring as indicators of the rapid evolution ongoing in telemedicine.

## New concepts in the treatment of atrial fibrillation

Persistent AF is understood by far to be the most challenging atrial arrhythmia to be treated by ablation. In the electrophysiology community, there is a clear consensus that the achievement of pulmonary vein (PV) isolation (PVI) is not adequate enough to treat patients suffering from persistent AF. In this regard, the location of so-called “non-PV triggers” is considered of pivotal significance in order to improve the success rate of ablation. To this end, Dr. Francis Marchlinski offered a significant contribution to the understanding of the role of non-PV triggers with his presentation, which highlighted the concept that, if PVs are isolated and there is still AF, there therefore must be non-PV triggers located elsewhere in the atria.^1^ He defined the protocols for initiating non-PV triggers, ranging from an infusion of incremental doses of isoproterenol up to 30 mcg/min to the use of burst pacing during isoproterenol infusion and adenosine administration. Prior research suggests that non-PV triggers are more frequently induced in females and more often found in the left atrium (> 70% of cases) than in the right atrium, with site locations including the crista terminalis (CT), the coronary sinus, in the antra of PVs, and the left atrial appendage (LAA).^2,3^ One understood clue for unmasking triggers is to position multipolar catheters in different locations such as along the CT, LAA, and in the coronary sinus. Most important is the validation of the value of non-PV trigger elimination by repeating the provocation protocol. This approach, by ensuring the elimination of non-PV triggers, can significantly improve the clinical outcome as compared with in patients in whom non-PV triggers are not eliminated during the redo procedure. Dr. Marchlinski alluded to this in his presentation, stressing that the occurrence of non-PV triggers at the repeat procedure identifies a poorer outcome.

Another extensively discussed topic was LAA electrical isolation in patients with long-lasting persistent AF, covered by Dr. Luigi Di Biase.^4^ This remains a hot topic in electrophysiology, since it appears that secondary foci can be identified in the LAA in about 20% to 25% of patients with persistent AF,^5^ and prior clinical data suggest that the procedural outcome is better when the LAA is electrically isolated even during the index procedure.^5^ The rationale supporting LAA isolation relies on the thought that the prevalence of non-PV triggers (and therefore also the likelihood of the LAA being a source of secondary triggers) is higher in patients with long-lasting persistent AF. Furthermore, the LAA hosts cells with automaticity, which can remain active even after extensive atrial substrate ablation and PVI, leading to a greater chance for arrhythmia recurrence. Clinical data from different groups have demonstrated the reproducibility of LAA isolation, providing evidence that this approach can play a role in improving the clinical outcome of patients with persistent AF. In this regard, a recent meta-analysis by Romero et al.^6^ corroborated the suggestion that LAA isolation constitutes a clinical benefit when included in the ablative strategy for patients with persistent AF.

The safety of catheter ablation of AF remains of pivotal importance and was discussed by Dr. David Haines, who also pointed out the subtle risk of promoting microemboli during radiofrequency (RF) delivery in the left atrium.^7^ In his presentation, Dr. Haines explored the subject in question, underlying the main biophysical aspects of RF current and stressing that it is important to pay full attention to anticoagulation, sheath management, and the unique features of the ablation technology being employed in each case. Probably the most striking results that Dr. Haines presented were related to the potential neuropsychological effects and the subtle cognitive dysfunction that can be detected after AF ablation. Specifically, reduced brain dysfunction, as demonstrated with brain magnetic resonance imaging, is more pronounced in the first six months after ablation, but progressively lessens over time.^8^ Again, the main cause of this finding was the promotion of microemboli formation during RF delivery, which favors the occurrence of asymptomatic cerebral lesions. In order to minimize the occurrence of asymptomatic cerebral emboli, Dr. Haines suggested that he supports the exploration of a multifaceted protocol that includes a proper transseptal technique, sheath flushing, optimal anticoagulation, avoidance of overpowered ablation, and possibly the use of improved ablation algorithms and catheter designs.

## News from the late-breaking clinical trials

As always, the late-breaking clinical trials sessions are ripe with intriguing data. From among them, for this commentary, I sought to focus on the potential role of vagus nerve stimulation to suppress AF, which has been extensively investigated in the last few decades and which remains a controversial topic. During one session, Dr. Stavros Stavrakis offered up the latest results of the Transcutaneous Electrical Vagus Nerve Stimulation to Suppress Atrial Fibrillation (TREAT-AF) randomized clinical trial, where 53 patients with paroxysmal AF were subdivided into two arms: an active group receiving vagus nerve stimulation at the tragus and a sham group receiving earlobe stimulation.^9^ The study in question demonstrated that, after combining the results recorded at the three- and six-month follow-up points, the median AF burden was reduced by 75% in the active group as compared with in the sham group. These impressive clinical results support the validity of pursuing further exploration of noninvasive neuromodulation to treat AF.

In the scenario of catheter ablation, one of the main challenges is by far the successful ablation of ventricular arrhythmias, including—in particular—so-called intractable VT and premature ventricular contractions (PVCs). In this regard, specific interest has grown regarding the use of intramural infusion needle to ablate deep localizations of ventricular rhythms. Dr. Tomofumi Nakamura presented preliminary data on the application of this technique in 19 patients with PVCs/VTs with deep origins in the septum or left ventricular summit.^10^ All of these patients had undergone a previous failed ablation attempt, and the infusion needle ultimately produced acute arrhythmia elimination in 68% of patients, with the arrhythmia burden reduced from 24.2% ± 8.5% to 2.7% ± 5.4% and the left ventricular ejection fraction improved from 32.4% ± 11.4% to 42.4% ± 6.8%, respectively. These preliminary clinical data suggest the technique presented is a promising ablative therapy for the management of intramural ventricular arrhythmias that have failed standard catheter ablation.

The search for an effective energy source to achieve durable lesions in AF ablation has been an attractive goal for several investigators in the last few decades, and the time for the availability of an alternative energy option to RF current has come. Pulsed electric-field ablation (PFA) has been investigated for a number of years to date and now finally seems ready to be employed in the clinical setting. The technology promotes cell death through the nonthermal mechanism of irreversible electroporation. During a late-breaking clinical session at HRS 2019, Dr. Jacob Koruth discussed the preliminary data for endocardial mono- or biphasic PFA for PVI in a swine model.^11^ The study demonstrated comparable chronic PVI rates for the two PFA waveforms, but biphasic PFA did not require neuromuscular paralysis as was necessary with the monophasic approach, and this constitutes a basis for possible clinical use of the biphasic PFA waveform in humans. Furthermore, since myocardial cells are particularly sensitive to PFA as opposed to other organs like the esophagus, larger vessels, and nerves, the use of this disruptive technology seems to be highly promising for specific deployment in humans, since potential complications are expected to be limited.

Cryoenergy has become an established effective alternative energy source for the ablation of AF, in part because it is less likely to adversely impact areas surrounding the target (i.e., cooling effect is more reversible) and may be less painful than RF ablation. To this end, data on the use of an ultra-low-temperature cryoablation system were presented by Dr. Felix Bourier, who discussed the safety, efficacy, and long-term durability results of using a preshaped catheter to deliver ultra-low cryoenergy in a porcine model.^12^ This technique reportedly led to effective lesion formation both in the atria and in the ventricles as demonstrated by extensive three-dimensional mapping and histopathology. More striking was the finding that the lesions were confirmed even at a 90-day reevaluation point in animals that underwent three-dimensional remapping and histopathology. No major complications occurred. The study authors concluded that this novel modality of cryoenergy application might be safely deployed in humans to attain a higher degree of lesion durability.

## New avenues in cardiac pacing

His-bundle pacing has been a hot topic in the last few years and, during HRS 2019, there were several presentations on the subject. Of note, a poster by Zanon et al.^13^ suggested reducing the minimum value of the pacing threshold along with performing a simultaneous optimization of the sensing capability of the apical back-up lead. This approach ultimately led to an energy consumption savings of 24%, thus increasing the device longevity by 32%. To counteract potential skepticism about the role of His-bundle pacing, another poster^14^ discussed a long-term follow-up (more than five years) of more than 400 patients, finding effective, persistent His-bundle pacing was successful in 81% of them and a requirement for reintervention in the same time period only occurred in 5.5%.

Also in the field of pacing, the talk given by Dr. Petr Neuzil about the current status of leadless implantable cardioverter-defibrillator (ICD) and cardiac resynchronization therapy (CRT) technology was of great interest.^15^ With the advent of the leadless pacemaker, a future goal has evolved into being, which is to devise a technology able to provide both effective ICD intervention and a CRT effect. Dr. Neuzil discussed the latest results obtained from a recent study in this area,^16^ which demonstrated a degree of feasibility for establishing a reliable communication between a current leadless pacemaker and an extravascular ICD, offering the capability for not only effective shock delivery/antitachycardia pacing but also accurate monitoring of the cardiac rhythm after a shock is delivered. Additionally, efforts are still ongoing to actualize a full leadless ICD/CRT device: this is the focus of the Wireless Stimulation Endocardially for CRT (WiSE-CRT) study,^17^ which includes different components: a coimplanted leadless right ventricular pacemaker; a receiver electrode implanted in the left endocardium; a battery implanted subcutaneously in the left mid-axillary line, and a phased ultrasound transmitter implanted submuscularly using cardiac echocardiography. Briefly, the system, via the submuscular transmitter, provides synchronization with the right ventricular pacing pulse to transmit ultrasound energy to the left endocardial receiver to provide biventricular pacing. The aim with this device was to avoid the use of any intravascular components so as to reduce complications such as infections, lead malfunction, and issues with surgically created pockets that currently still constitute major confounding factors in the management of intravascular devices.

## Remote arrhythmia detection: state-of-the-art and future opportunities

Last but not least, the subject of adopting novel wearable devices for detecting cardiac arrhythmia disorders including specifically AF was one of focus.^18^ This interest is related to the continued desire to address the overwhelming burden that cardiac disorders can produce through the introduction of newer and more innovative technologies. In this regard, providers must understand key concepts in electrophysiology science and management, including the effectiveness and complications of the diagnosis and how to manage patients appropriately. Therefore, the advent of new diagnostic tools such as wearable devices and watches are crucial to encourage an open discussion of guidelines, perceived challenges, and future directions in clinical practice to optimize patient care. The different symposia on this hot topic were well-designed for the physicians and allied health care providers alike who are in charge of managing arrhythmic patients who are also at risk of comorbidities. Remote diagnosis of cardiac arrhythmia disorders and better communication among patients, physicians, and other health care providers will become more crucial in the years to come and will be an important step in improving patient care.

## References

[1] Marchkinski F (2019). Non PV triggers: approach to mapping and ablation. Presented at: Heart Rhythm Society Scientific Sessions; May 8.

[2] Hojo R, Fukamizu S, Kitamura T (2017). Development of nonpulmonary vein foci increases risk of atrial fibrillation recurrence after pulmonary vein isolation.. JACC Clin Electrophysiol..

[3] Takigawa M, Takahashi A, Kuwahara T (2015). Impact of non-pulmonary vein foci on the outcome of the second session of catheter ablation for paroxysmal atrial fibrillation.. J Cardiovasc Electrophysiol..

[4] Di Biase L (2019). Appendage isolation for AF management: where do we stand today? Presented at: Heart Rhythm Society Scientific Sessions; May 9.

[5] Di Biase L, Burkhardt JD, Mohanty P (2010). Left atrial appendage: an underrecognized trigger site of atrial fibrillation.. Circulation..

[6] Romero J, Michaud GF, Avendano R (2018). Benefit of left appendage electrical isolation for persistent and long-standing persistent atrial fibrillation: a systematic review and meta-analysis.. Europace..

[7] Haines D (2019). Thromboembolic complications of AF ablation: asymptomatic cerebral emboli. Presented at: Heart Rhythm Society Scientific Sessions; May 8.

[8] Herm J, Fiebach JB, Koch L (2013). Neuropsychological effects of MRI-detected brain lesions after left atrial catheter ablation for atrial fibrillation: long-term results of the MACPAF study.. Circ Arrhythm Electrophysiol..

[9] Stavrakis S, Stoner JA, Humphrey MB (2019). Transcutaneous electrical vagal nerve stimulation to suppress atrial fibrillation (TREAT-AF): a randomized clinical trial. Abstract presented at: Heart Rhythm Society Scientific Sessions; May 9.

[10] Nakamura T, Dukkipati SR, Nakajima I (2019). Intramural infusion needle ablation for intractable premature ventricular contractions associated with ventricular dysfunction. Abstract presented at: Heart Rhythm Society Scientific Sessions; May 9.

[11] Koruth J, Kuroki K, Iwasawa J (2019). Durability of PV isolation with pulse electric field ablation: a pre-clinical comparison of monophasic and biphasic waveforms. Abstract presented at: Heart Rhythm Society Scientific Sessions; May 9.

[12] Bourier F (2019). Novel ultra-low temperature cryoablation system: safety, efficacy and long-term durability of lesions in a porcine model. Abstract presented at: Heart Rhythm Society Scientific Sessions; May 8.

[13] Zanon F, Marcantoni L, Pastore G (2019). Pacing impulse optimization in His bundle pacing. Poster presented at: Heart Rhythm Society Scientific Sessions; May 11.

[14] Zanon F, Marcantoni L, Zuin M (2019). System-related surgical reinterventions in His bundle pacing. Poster presented at: Heart Rhythm Society Scientific Sessions; May 9.

[15] Neuzil P (2019). The current state of leadless ICD and CRT! Presented at: Heart Rhythm Society Scientific Sessions; May 9.

[16] Tjong FVY, Brouwer TF, Koop B (2017). Acute and 3-month performance of a communicating leadless antitachycardia pacemaker and subcutaneous implantable defibrillator.. JACC Clin Electrophysiol..

[17] Auricchio A, Delnoy PP, Butter C (2014). Feasibility, safety, and short-term outcome of leadless ultrasound-based endocardial left ventricular resynchronization in heart failure patients: results of the wireless stimulation endocardially for CRT (WiSE-CRT) study.. Europace..

[18] Loveless K (2019). Strategies to use mobile medical technology to enhance clinician–patient communication. Presented at: Heart Rhythm Society Scientific Sessions; May 9.

